# Electro–opto–mechano driven reversible multi-state memory devices based on photocurrent in Bi_0.9_Eu_0.1_FeO_3_/La_0.67_Sr_0.33_MnO_3_/PMN-PT heterostructures†

**DOI:** 10.1039/d0ra00725k

**Published:** 2020-04-22

**Authors:** Maocai Wei, Meifeng Liu, Lun Yang, Xiang Li, Yunlong Xie, Xiuzhang Wang, Zijiong Li, Yuling Su, Zhongqiang Hu, Jun-Ming Liu

**Affiliations:** School of Physics and Electronic Engineering, Zhengzhou University of Light Industry Zhengzhou 450002 China; Institute for Advanced Materials, Hubei Normal University Huangshi 435002 China; Electronic Materials Research Laboratory, Key Laboratory of the Ministry of Education & International Center for Dielectric Research, Xian Jiaotong University Xian 710049 China; Laboratory of Solid State Microstructures, Nanjing University Nanjing 210093 China

## Abstract

A single device with extensive new functionality is highly attractive for the increasing demands for complex and multifunctional optoelectronics. Multi-field coupling has been drawing considerable attention because it leads to materials that can be simultaneously operated under several external stimuli (*e.g.* magnetic field, electric field, electric current, light, strain, *etc.*), which allows each unit to store multiple bits of information and thus enhance the memory density. In this work, we report an electro–opto–mechano-driven reversible multi-state memory device based on photocurrent in Bi_0.9_Eu_0.1_FeO_3_ (BEFO)/La_0.67_Sr_0.33_MnO_3_ (LSMO)/0.7Pb(Mg_1/3_Nb_2/3_)O_3_-0.3PbTiO_3_ (PMN-PT) heterostructures. It is found that the short-circuit current density (*J*_sc_) can be switched by the variation of the potential barrier height and depletion region width at the Pt/BEFO interface modulated by light illumination, external strain, and ferroelectric polarization reversal. This work opens up pathways toward the emergence of novel device design features with dynamic control for developing high-performance electric–optical–mechanism integrated devices based on the BiFeO_3_-based heterostructures.

## Abstract 2

The mutual interaction between polarization switching, light and piezoelectric strain.

## Graphical abstract

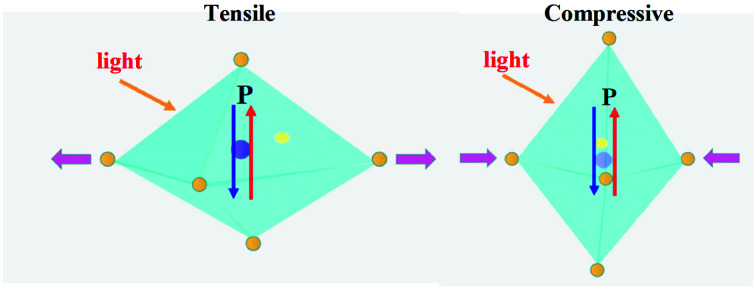

## 1. Introduction

Facing the challenge of the limitation of Moore's law,^1,2^ a single device that integrates extensive new functionality is highly desired for the ever-increasing demands for devices with faster, higher density, high energy efficiency data processing/storage with reversible and nonvolatile manipulation.^3–5^ Very recently, multiferroic materials and heterostructures have been reported as two of the most promising candidates for realizing such an integration of different functions.^6–12^ However, most of the studies mainly focus on the functional controllability using an external electric or magnetic field, rarely paying attention to exploration of other external control parameters. In the quest for multifunctional materials, interactions arising from different media or dimensions are highly desirable. Nowadays, light control has become a novel manner with nondestructive program/erase process compared with electrical tuning, and can enhance the device performance remarkably.^2,13^ These processes can be achieved through transforming light signal to electric response so as to achieve multi-bit and encryption storage. More particularly, light could also solve sneak path currents that plague ultralow cost electronics,^4,14^ which endows new functionality for memory devices and may open possibilities of remote control for advanced optoelectronic applications.

BiFeO_3_ (BFO) is a multiferroic materials with robust ferroelectric and magnetic orders above room temperature^15–17^ and a narrow optical bandgap (∼2.5 eV)^18,19^ than traditional ferroelectric photo-voltaic materials (such as LiNbO_3_,^20^ BaTiO_3_,^21^ (Pb,Zr)TiO_3_,^22^ with quite large bandgap *E*_g_ > 3.0 eV). It offers considerable light absorption capability and endows with novel physics and new degrees of freedom for multifunctional devices. However, many efforts have been devoted to enhancing the photovoltaic (PV) of BFO,^18,23–28^ but unfortunately the power conversion efficiency is still poor (<0.2%) compared with other types of solar cells.^28^ Thus, until recently ferroelectric photovoltaic (FE-PV) effect is still in its infancy rather than any real application. Alternatively, not just limited in the power conversion efficiency, the signal of photovoltaic effect can allow for information transfer and storage applications.^29,30^ A unique characteristic of FE-PV devices is that the photocurrent direction can be switched by reversing the spontaneous polarization direction of a FE material with electric field.^18^ This feature is compatible and of central importance for the development of on-chip optical communication technology.

More significantly, since Choi *et al.* first reported the switchable ferroelectric diode and visible light-induced photovoltaic effects in BFO single crystals,^23^ the coupling of ferroelectric polarization with optical properties in photo-active ferroelectrics has received a renewed attention, triggered notably by low bandgap ferroelectrics and original photovoltaic effects.^31–34^ Interestingly, experimental and theoretical investigations have evidenced that the bandgap and polarization of a perovskite-type ferroelectric thin film can be tuned by substrate-induced strain.^31–34^ Fu *et al.* realized a maximum bandgap shift of 0.7 eV by growing BFO films on SrTiO_3_ substrates with various in-plane compressive strain.^33^ Sando *et al.* reported that BFO films deposited on different substrates can accommodate different kinds of strains from compressive one to tensile one.^34^ However, these strain control schemes cannot rule out the effects of other extrinsic factors (oxygen non-stoichiometry, defects, dead layer, disorder, *etc.*) on the photovoltaic effects.

Alternatively, ferroelectric Pb(Mg_1/3_Nb_2/3_)O_3_-PbTiO_3_ (PMN-PT) single crystals are well known for their ultrahigh piezoelectric coefficient, large electromechanical coupling, and excellent ferroelectric and pyroelectric responses.^35^ Numerous experimental works have demonstrated that the in-plane strain of oxide films epitaxially grown on (1 − *x*)Pb(Mg_1/3_Nb_2/3_)O_3_-*x*PbTiO_3_ (0.28 ∼ *x* ∼ 0.33) ferroelectric substrates can be *in situ* and dynamically manipulated by applying an electric field to the PMN-PT substrates, and thus the aforementioned various influences can be avoided to a great degree.^10–12,35–39^

Along this line, an emergent scheme is to fabricate multifunctional devices that can be achieved by combining a series of monofunctional components into a tight space.^40^ However, as monofunctional devices mature and approach their fundamental limits, a pivotal question now is how to develop technologies enabled with increasing functionalities and reduced product size. A better and highly concerned scheme is to integrate multiple functionalities into one material for multi-parametric detection. If it applies, the device structure would be significantly simplified, benefiting to the fabrication complexity reduction, integration level boosting, and possible application expansion.

So far, a comprehensive understanding of the combined electric-field, light, and mechanical control of FE-PV and the resultant functionalities, both *in situ* and dynamically, are still limited for BFO thin films. Moreover, there is an urgent demand for optoelectronics devices with a multifunctional integration and minimal size, which can satisfy the demand for fast, high density, high energy efficiency data processing/storage. There is no doubt that a systematic investigation of multi-field tuned physical properties of BFO thin films is highly expected. In this work, we report an electro–opto–mechano-driven reversible and multi-state memory device, based on photocurrent effect in Bi_0.9_Eu_0.1_FeO_3_ (BEFO)/La_0.67_Sr_0.33_MnO_3_ (LSMO)/PMN-PT heterostructures. The main property to be targeted for such a multi-field tuning is the photo-induced electronic conduction. For a high-performance consideration, we focus on BEFO thin films rather than BFO simply due to the fact that a slight Eu substitution at the Bi site would benefit to the ionic defect and leakage suppression.

Our comprehensive investigation suggests that the photo-current *J*_sc_ of our heterostructures can be effectively switched by modulating the potential barrier height and depletion layer width at the Pt/BEFO interface, by means of various stimuli such as light illumination, strain, and ferroelectric polarization. More importantly, the device can produce photocurrent that allows us to read out the state in a self-powered manner. This discovery provides the means for engineering tunable single memory unit but enabling multi-functionalities such as switching between the volatile and nonvolatile modes by electric–optical–mechanical combination mode. This mode and associated functionalities open the door to novel multifunctional storage devices.

Herein, we propose a memory micro-array based on the Pt/BEFO/LSMO/PMN-PT heterostructures to demonstrate our idea. In this prototype, “0” state and “1” state represents the P_up_ state and P_down_ state of the BEFO film layer, respectively. The writing of “0” or “1” state in each cell can be easily realized by reversing the spontaneous polarization direction of the BEFO film with electric field. During reading the data, an incident light is irradiated to the whole micro-array, meanwhile, the induced *J*_sc_ is directly detected for each cell at a zero-bias. Moreover, different polarization state produces different *J*_sc_, the data state in each cell is thus read out. This data storage mode can be called as “electrical writing and optical reading”, which consumes fewer power than conventional modes. We can also develop a logical switching device that a unit cell with P_up_ (or P_down_) state, its logic state will be alternately switched to ON or OFF state when the incident light is on or off, then be read out by detecting the corresponding *J*_sc_. What's more, this kind of electronic device has much room to improvement when the light was turned on and off with and without a certain strain applied to the unit cell, which could get more reliable, larger/smaller, and switchable *J*_sc_.

## 2. Experimental

The LSMO and BEFO thin films were epitaxially grown on the (011)-oriented but non-poled PMN-PT substrates using the pulsed laser deposition (PLD) technique. First, the LSMO layer with thickness of 60 nm was deposited on the substrate with a deposition rate of 4 nm min^−1^, where the temperature and laser repetition ratio were kept at 700 °C and 3 Hz under an oxygen pressure of 40 Pa. After the deposition, the oxygen was filled into the chamber at maximal flow rate and the as-grown LSMO was annealed *in situ* for 30 min at 700 °C and then cooled down to room temperature at 4 °C min^−1^. Subsequently, the BEFO layer with thickness of 120 nm was deposited on the LSMO thin film at a deposition rate of 2 nm min^−1^, where the temperature and laser repetition ratio were kept at 660 °C and 3 Hz under an oxygen ambient pressure of 15 Pa. Afterwards, the oxygen was filled into the chamber at maximal flow rate right after the deposition, while the sample was cooling at a rate of 2 °C min^−1^. When the chamber pressure reached 1.0 atm, the thin film was annealed at 400 °C for 30 min and then cooled to room temperature at 4 °C min^−1^. In the case of precise control of above parameters, the film thickness is controlled by the number of laser pulses.

**Fig. 1:**
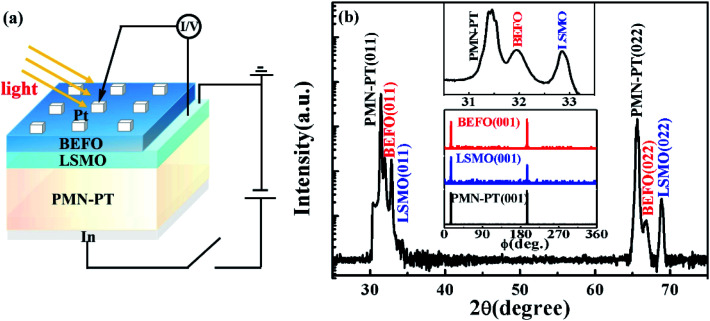
(a) Schematic of Pt/BEFO/LSMO/PMN-PT heterostructures. (b) XRD *θ*–2*θ* scan of the heterostructures, and the inset (up) is the expanded view near BEFO (011) diffraction peak, the inset (down) is *ϕ*-scan of the BEFO (011) and NSTO (011) planes.

The structure of the as-prepared BEFO/LSMO/PMN-PT heterostructure was characterized by X-ray diffraction (XRD, Bruker D8 Advance). The ferroelectric domain structures were probed by the piezoresponse force microscopy (PFM, Bruker Multimode 8) using the Pt-coated Si cantilevers. For the strain control, we applied electric field across the piezoelectric PMN-PT substrate and induced the in-planar lattice distortion (strain) which was transferred into the BEFO thin film above the LSMO bottom electrode. A voltage source was employed to supply an electric field across the PMN-PT substrate through the LSMO bottom electrode and the In back-electrode on the back side of PMN-PT substrate (as shown in Fig. 1(a)). It is noted that the positive bias was applied to the In electrode and the ground was applied to the LSMO electrode in our measurement.

For the transport behaviors of the as-prepared BEFO thin film, the current–voltage (*J*–*V*) curve was measured across the BEFO film using the Keithley 4200 characterization system with the voltage sweeping mode. Here, the top Pt electrode with a 0.5 mm × 0.5 mm square shadow mask was deposited on the BEFO film surface using the magnetron sputtering technique. The positive (negative) voltage was defined as the positive (negative) bias applied to the top Pt electrodes.

For the light illumination, a diode laser with a wavelength of 405 nm (*hv* = 3.06 eV) and power density of 200 mW cm^−2^ was used as the illumination source. The measurement setup schematic is shown in Fig. 1(a), where photo-current *J*_sc_ was obtained using the following mode. First, the sample was poled by a voltage pulse *V*_P_ of 100 ms in width in the dark or light illumination condition. Second, the sample was submitted to a low voltage sweeping from −0.5 V to 0.5 V during which the *J*–*V* curve was obtained, noting that a voltage of ±0.5 V is insufficient to change the initial polarization state. Third, the value of *J*_sc_ was extracted from the *J*–*V* curve at *V* = 0. Certainly, *J*_sc_ can be also directly probed in the illumination ON/OFF testing (*J*_sc_ = 0 in the OFF case).

## 3. Results and discussion

### 3.1. Structural characterizations

Fig. 1(b) shows the *θ*–2*θ* scan XRD pattern of the as-prepared heterostructure grown on PMN-PT (011) substrates, in which only strong (0*ll*) (*l* = 1, 2) diffractions from the BEFO and LSMO thin films and substrate appear. It is suggested that both the BEFO and LSMO films are single phase and highly (011)-oriented. The expanded view near the BEFO (011) diffraction peak is shown in the inset (up) while the inset (down) exhibits the *φ* scan of the (001) plane. The two-fold symmetrical diffraction peaks from the BEFO and LSMO films can be clearly identified and they occur at the same *φ* angles as those of the PMN-PT substrates, confirming a cube-on-cube epitaxial growth of the BEFO/LSMO films on PMN-PT substrates. From the XRD spectra, the out-of-plane (OP) lattice constants of the BEFO, LSMO, and PMN-PT, as calculated from the (011) diffraction peak are 2.797, 2.724, and 2.842 Å, respectively.

A further discussion on the strain state in the BEFO thin film begins by looking at the lattice mismatch of 1.58% between the film and substrate. The BEFO film has smaller OP lattice constant than that of bulk BFO (∼2.80 Å),^41^ revealing that the BEFO film is subjected to an OP compressive strain (∼−0.11%). Similarly, the OP lattice constant of the LSMO film is smaller than that of bulk LSMO (∼2.736 Å),^42^ revealing that the LSMO film is subjected to an OP compressive strain (∼−0.44%). According to the Poisson equation, the OP strain can be deduced as *ε*_OP_ = −2*νε*_IP_/(1 − *ν*), where *ε*_OP_ and *ε*_IP_ are the OP and in-plane (IP) strains and *ν* is the Poisson's ratio, given the condition of constant unit cell volume. The Poisson's ratios for the BEFO and LSMO films are ∼0.34 (ref. 43) and ∼0.37 (ref. 44), respectively. Thus, the estimated IP tensile strains for the BEFO and LSMO films are ∼0.10% and ∼0.37%, respectively. These data suggest that the lattice strain in the PMN-PT substrate could largely be coherently transferred into the BEFO film.

### 3.2. Ferroelectric behaviors

**Fig. 2:**
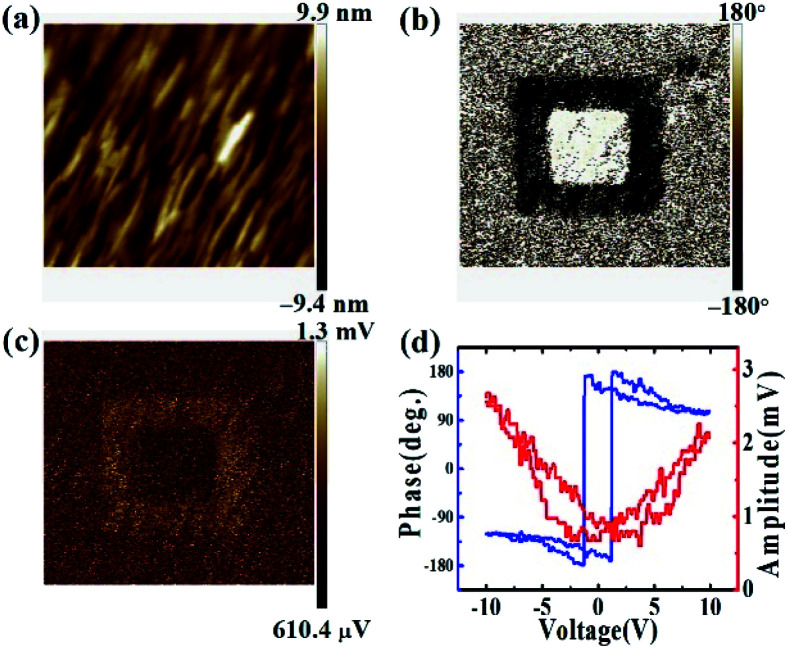
(a) Topological, (b) PFM phase and (c) amplitude images of the of BEFO film after applying +10 V (inner square 0.6 μm × 0.6 μm) and −10 V (outer square 1.0 μm × 1.0 μm) bias at the center of the region, respectively. (d) PFM phase and amplitude hysteresis loops.

To characterize the ferroelectric properties of the as-prepared BEFO thin film, vertical piezoresponse force microscopy (V-PFM) was performed and the results are highlighted in Fig. 2. The atomic force microscopy on the BEFO film shows the small surface roughness of 2.16 nm (Fig. 2(a)). Using the PFM mode, a region of the BEFO film can be electrically switched to create a “box-in-box” pattern, as shown in Fig. 2(b) for the phase contrast and Fig. 2(c) for the amplitude contrast. The outer region with a scan size of 1.0 μm × 1.0 μm corresponds to the up-polarization (up-state) under a tip voltage of −10 V, while the internal region with a scan size of 0.6 μm × 0.6 μm corresponds to the down-polarization (down-state) state under a tip voltage of +10 V. Furthermore, the typical phase and butterfly-like amplitude loops are shown in Fig. 2(d) with the coercive voltages of about −1.3 V and 1.2 V, revealing the good ferroelectric property of the BEFO film.

The PFM phase imaging also clearly identified that the self-polarization of the fresh state is oriented upwards, which can be seen from the indistinct colour contrast between the ±10 V switching zone and the non-switching zone. This preference is believed to originate from the lattice mismatch induced residual stress in the thin film. It is noted that the fresh BEFO film was in the OP compressive state, leaving the positive and negative polarization charges to be created at the Pt/BEFO and BEFO/LSMO interfaces, as evidenced by earlier reports.^45^

### 3.3. Polarization control

**Fig. 3:**
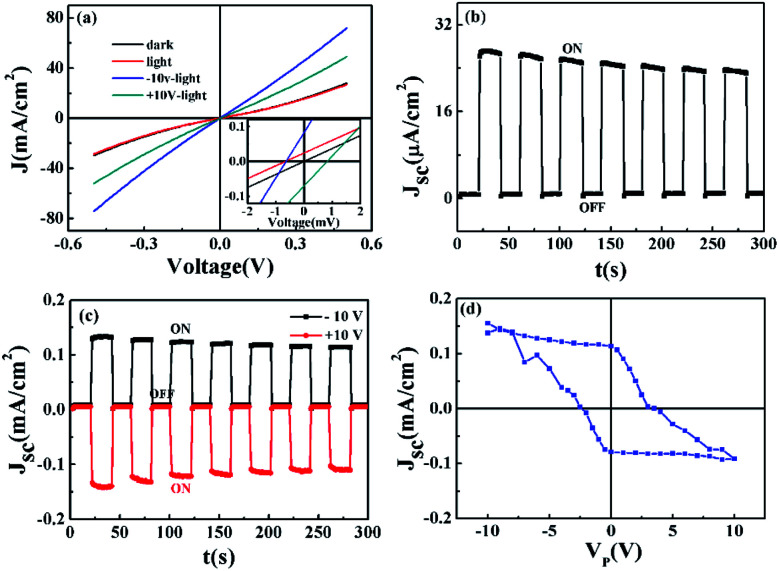
(a) *J*–*V* curves for the Pt/BEFO/LSMO/PMN-PT device in the dark and under light illumination (*λ* = 405 nm) where the BEFO film was in the unpoled (P_r_^0^), positively poled (P_r_^+^), and negatively poled (P_r_^−^) states, respectively, and the PMN-PT was in the unpoled state. The inset shows the expanded region of the *J*–*V* curves. (b) The *J*_sc_ as a function of time for the Pt/BEFO/LSMO/PMN-PT device where the BEFO was in the P_r_^0^ state. (c) The *J*_sc_ as a function of time upon turning the light on and off for the Pt/BEFO/LSMO/PMN-PT device where the BEFO film was in the P_r_^+^ and P_r_^−^ states, respectively. (d) *J*_sc_ as a function of the voltage pulse (*V*_P_) under light illumination.

The photovoltaic responses of the BEFO film were characterized by measuring the *J*–*V* curves under the light illumination (*λ* = 405 nm) onto the unpoled BEFO thin film with remnant polarization P_r_^0^, positively poled film with remnant polarization P_r_^+^, and negatively poled film with remnant polarization P_r_^−^, respectively. It is noted that in this experiment, the PMN-PT substrate was in the unpoled state and thus no substrate polarization induced strain is available. The measured data are plotted in Fig. 3(a), from which several features deserve highlighting.

First, at zero bias, no open-circuit voltage *V*_oc_ and no short-circuit current *J*_sc_ can be observed in the dark, as shown in Fig. 3(a). However, the BEFO film under the light illumination shows significant photovoltaic responses with *V*_oc_ = −0.7 mV and *J*_sc_ = 24.4 μA cm^−2^, suggesting that the built-in electric field can be affected by the illumination due to the photo-generated charges. Such an effect shows good retention, supported by the modulated photocurrent *J*_sc_ as a function of time *t* by the light ON/OFF operation with an interval of 20 s, as shown in Fig. 3(b).

Second, to investigate the polarization-modulated photovoltaic effect, the BEFO layer in the heterostructure was pre-poled using a pulse of *V*_P_ = 10 V and −10 V (pulse width 100 ms) so that the BEFO film had the down- and up-polarization states, respectively, noting that the PMN-PT substrate remained to be unpoled. Here, the positive (negative) voltage is defined as the positive (negative) bias applied to the top Pt electrodes, and the positively and negatively poled states represent the up- and down-states, respectively. As shown in Fig. 3(a), the *V*_oc_ and *J*_sc_ values for the up-state increase up to −0.9 mV (by −28%) and 82.8 μA cm^−2^ (by 2390%), respectively. For the down-state with remnant polarization P_r_^−^, the measured *V*_oc_ and *J*_sc_ changed their signs and became 0.6 mV (by 186%) and −70.1 μA cm^−2^ (by −3870%), respectively.

Third, the measured *J*_sc_ shows the opposite responses for the thin film in the up-state and down-state, as more directly seen in Fig. 3(c), by turning the light ON/OFF with an interval of 20 s, respectively. Here, it is possible to determine the polarization direction (stored information) by sensing the value of *J*_sc_, and this process is non-destructive. Subsequently, we performed a series of measurements in which different pulse voltages (*V*_P_) to pole the BEFO thin film were set and the measured *J*_sc_–*V*_P_ hysteresis loop is plotted in Fig. 3(d). This loop exhibits hysteretic behaviors similar to the *P*–*V* loop shown in Fig. 2(d), indicating that the ferroelectric polarization of the BEFO thin film plays a key role in the switchable photovoltaic effect. The asymmetric *J*_sc_–*V*_P_ curves also suggest that the photovoltaic response was dominated by the polarization modulated interfacial barriers rather than the bulk photovoltaic effect (BPE),^46^ noting that the bulk effect dominated effect would produce a symmetric *J*_sc_–*V*_P_ curve.

### 3.4. Strain control

**Fig. 4:**
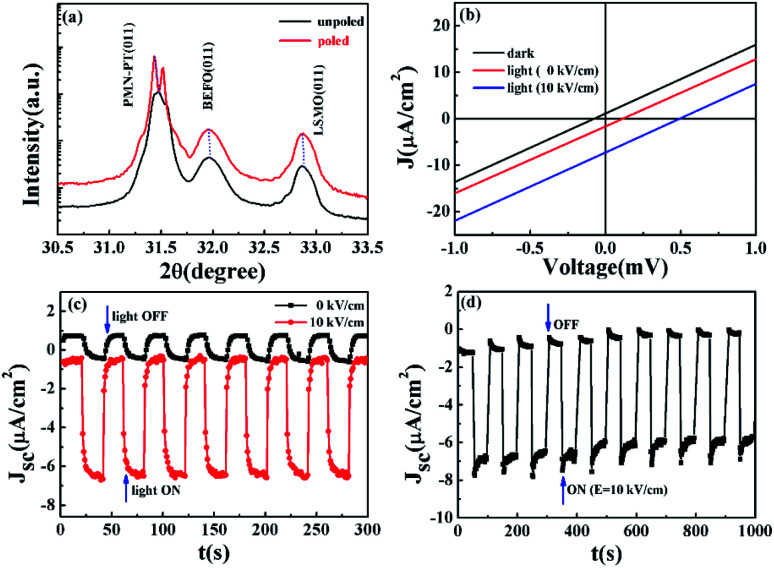
(a) XRD *θ*–2*θ* scan patterns near PMN-PT (011) and BEFO (011) diffraction peaks under unpoled and poled states to the PMN-PT. (b) *J*–*V* curves for the Pt/BEFO/LSMO/PMN-PT device in the dark and under light illumination with and without the application of an electric field of +10 kV cm^−1^ to the PMN-PT substrate for the Pt/BEFO/LSMO/PMN-PT device where the BEFO was in the unpoled state while the PMN-PT was in the positively poled state. (c) The *J*_SC_ as a function of time upon turning the light on and off for the Pt/BEFO/LSMO/PMN-PT device with and without the application of an electric field of +10 kV cm^−1^ to the PMN-PT substrate, where the BEFO film was in the P_r_^0^ state. (d) *J*_SC_ under light illumination (*λ* = 405 nm) as a function of time when the electric field of *E* = +10 kV cm^−1^ was turned on and off.

The previous subsections have been devoted to the modulation of photovoltaic effect by external electric field and ferroelectric polarization from the BEFO film. This section is devoted to the intrinsic effect of electric-field-induced PMN-PT piezoelectric strain on the photocurrent. To achieve it, a large dc electric field *E* ∼ 10 kV cm^−1^ across the PMN-PT substrate was applied for 30 min to ensure a full polarization state (*i.e.* the pole state) of the substrate, noting that the positive bias was applied to the In back-electrode and the negative bias to the LSMO electrode. Fig. 4(a) shows the *in situ* XRD data from the heterostructure, given the unpoled and poled states of the PMN-PT substrate. An expansion along the OP direction associated with an effective IP lattice contraction can be identified. It is thus revealed that the BEFO film is subjected to an OP compressive strain (∼−0.04%) and an IP tensile strain (0.03%), noting that the initial OP and IP strains of the BEFO thin film are −0.11% and 0.10%. The application of an electric field with the same direction as the poling direction would induce an IP compressive strain in the PMN-PT *via* the well-known converse piezoelectric effect.^47^ The induced strain is expected to be transferred to the BEFO film *via* the LSMO layer.

As a result of the substrate piezoelectric strain, the photovoltaic properties can be modified due to the variation of strain state inside the BEFO film. Fig. 4(b) shows the measured *J*–*V* curves of the BEFO film in the dark and under light illumination (*λ* = 405 nm), given that the PMN-PT substrate was in the unpoled and poled states respectively for a comparison. In this experiment, the BEFO film was in the unpoled state with remnant polarization P_r_^0^ in this measurement. Obviously, the *J*–*V* curve of the fresh BEFO film does not exactly pass through the origin in the dark, as shown in the inset of Fig. 3(a), indicating a built-in electric field in the heterostructure. Under the light illumination, the fresh BEFO film showed significant photo-voltaic response, also an indirect evidence for the built-in electric field.^48^ The measured *J*_sc_ ∼ −1.6 μA cm^−2^ and *V*_oc_ ∼ 0.1 mV were obtained, respectively, and the sign changes of *V*_oc_ and *J*_sc_ suggest that the built-in electric field can be affected by the illumination due to the photo-generated charges.^30,49^ The observed zero-bias photocurrent density was −1.6 μA cm^−2^, which is superior to most active ferroelectric oxide BiFeO_3_ (∼0.4 μA cm^−2^).^50^ Moreover, the M − H loops at different poling states have shown (Fig. S1†) that the magnetization of the heterostructure can be manipulated by the electric field induced piezo-strain, which could be used as evidence for magnetoelectric coupling effect at room temperature. However, we may need further research to understand this effect in depth.

Now we come to see the consequence of applying an electric field of +10 kV cm^−1^ to the PMN-PT substrate. The measured *V*_oc_ and *J*_sc_ significantly increased up to 1.8 mV and −7.2 μA cm^−2^, resulting in an enhancement by 1700% and 350%, respectively. These effects are much larger than earlier reported data for Fe_3_O_4_/PMN-0.29 PT (011) heterostructure where the modulation magnitude for magnetization is only <3% at 300 K and that for resistance is also only <3% at 300 K.^51^ Certainly, it is highly desirable to achieve a sufficiently large modulation of the concerned properties in a reversible and nonvolatile manner at room temperature, and thus the present work represents a substantial step to practical applications.

To check the stability and reliability of this piezoelectric strain induced effect, we measured *J*_sc_ as a function of time by turning the light ON and OFF, given that the PMN-PT substrate was under zero electric field and non-zero field *E* = +10 kV cm^−1^. The results are plotted in Fig. 4(c). It is seen that the measured *J*_sc_ under *E* = +10 kV cm^−1^ was much larger than that under *E* = 0. The magnitude of *J*_sc_ under *E* = +10 kV cm^−1^ is even more stable than the case under *E* = 0, upon turning the light on and off. These effects can be further demonstrated by turning ON/OFF the electric field applied to the PMN-PT substrate between *E* = +10 kV cm^−1^ and *E* = 0, given the on-state of the light illumination. As seen in Fig. 4(d), the sequence of *E* = +10 kV cm^−1^ and *E* = 0 did make the response of *J*_sc_ sharply and repeatedly. All these results demonstrate that the piezoelectric strain from the substrate can be used to modulate the photovoltaic effects in the present heterostructure.

### 3.5. Multi-field control

Given the photocurrent modulation using the ferroelectric polarization of the BEFO thin film and the piezoelectric strain from the substrate, it is time to investigate the multi-field control sequence. It is proposed that the control parameters include the polarization states (P_r_^+^ or ↑, P_r_^−^ or ↓) of the BEFO thin film and the piezoelectric strain states (1 @ *E* = 10 kV cm^−1^, 0 @ *E* = 0) of the PMN-PT substrate. The memory state of the Pt/BEFO/LSMO/PMN-PT heterostructure is characterized by the photocurrent *J*_sc_ stimulated by the light illumination on/off switching. Surely, we can reach at least eight photocurrent states that can be denoted by *J*_sc_(↑, 0, on), *J*_sc_(↓, 0, on), *J*_sc_(↑, 1, on), *J*_sc_(↓, 1, on), *J*_sc_(↑, 0, off), *J*_sc_(↓, 0, off), *J*_sc_(↑, 1, off), *J*_sc_(↓, 1, off), where the arrow ↑/↓ refers to the OP polarization state of the BEFO thin film, the number 0/1 refers to the off/on state of the electric field applied to the PMN-PT substrate, and the status on/off refers to the on/off light illumination state.

**Fig. 5:**
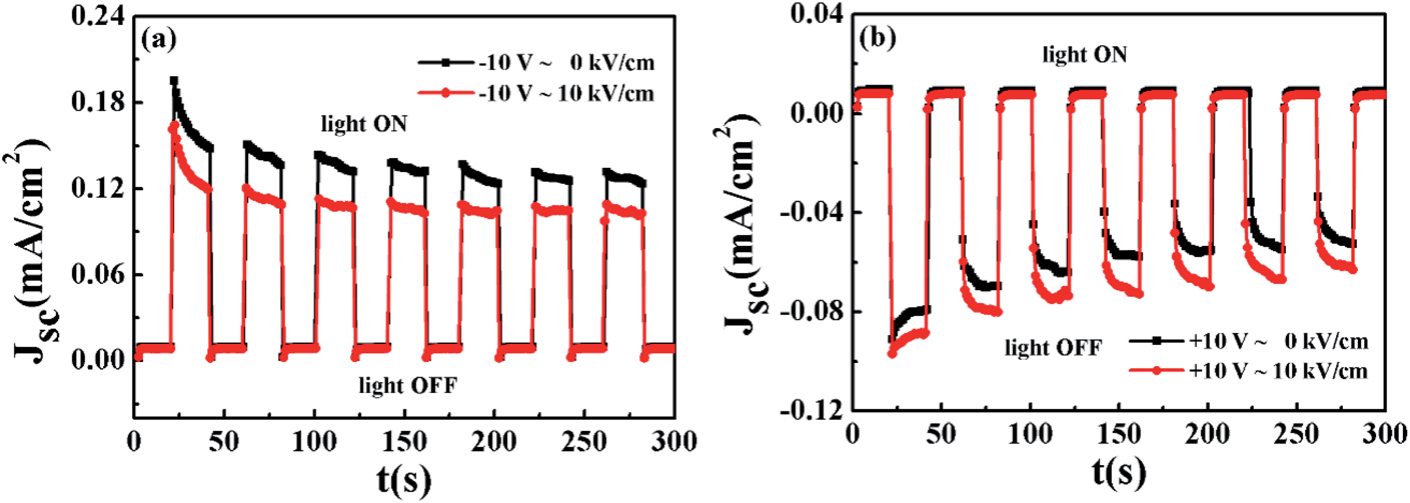
(a and b) *J*_sc_ switching of the BEFO film in the dark and under light illumination by switching the polarization. *J*_sc_ as a function of time upon turning the light on and off for the Pt/BEFO/LSMO/PMN-PT device with and without the application of an electric field of +10 kV cm^−1^ to the PMN-PT substrate, where the BEFO film was in the P_r_^+^ and P_r_^−^ states, respectively.

However, it should be mentioned that the difference in *J*_sc_ among the four light illumination off states is technically indistinguishable, and therefore usually the four states are treated as one high-resistance state (*J*_sc_ ∼ 0). In consequence, we can access the five states rather than eight states: *J*_sc_(↑, 0, on), *J*_sc_(↓, 0, on), *J*_sc_(↑, 1, on), *J*_sc_(↓, 1, on), *J*_sc_(off). Fig. 5 presents one set of measured data for multi-field control.

From Fig. 5(a and b), it is clearly shown that the five states can be easily accessed by simply switching on/off the light-illustration, the electric field applied to the PMN-PT substrate, and the ↑↓ ferroelectric polarization states. For a quantitative estimation of the photocurrent switching performance, one may define the equivalence-of-merit (EOM) as:1
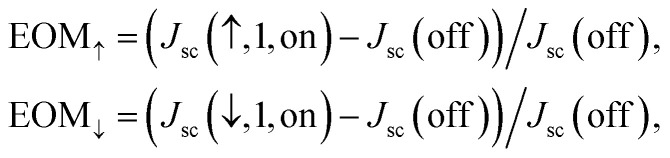


First, it is interesting to note that the EOM_↑_ and EOM_↓_ can be as large as ∼1130% and 1200%, respectively, representing the largest values so far reported in literature. Second, it is further demonstrated that the photocurrent switching on/off can be safely and reliably realized by either switching on/off the electric field applied to the PMN-PT substrate (piezoelectric switching on/off), or switching the ferroelectric polarization of the BEFO thin film between P_r_^+^ and P_r_^−^, or even switching both simultaneously. Whether with or without the application of *E* = +10 kV cm^−1^ to the PMN-PT, the signs of *J*_sc_ are always positive for P_r_^+^ and negative for P_r_^−^, respectively (as shown in Fig. 5 (a and b)). Moreover, when applying an electrical field of *E* = +10 kV cm^−1^ to the PMN-PT, the value of *J*_sc_ are reduced for P_r_^+^ and enhanced for P_r_^−^, respectively. Just looking at the data in Fig. 5 indicates that the light turn-on can make the magnitude of *J*_sc_ to rapidly rise up to 0.124 mA cm^−2^ and −0.088 mA cm^−2^ for the ↑ and ↓ states respectively, from the black state with *J*_sc_ ∼ 8.0 μA cm^−2^. Furthermore, sharp response to the on/off switching of the substrate piezoelectric strain is also clearly identified.

### 3.6. Discussion

In this section, we focus on the possible mechanisms underlying such multi-field control processes, and a comprehensive microscopic scenario would be very helpful for designing and fabricating advanced devices.

Obviously, once the piezoelectric strain is applied, the negative photocurrent (for P_r_^−^) will be enhanced, the positive photocurrent (for P_r_^+^) will be reduced. The underlying mechanism is dominated by the enhanced (or reduced) the barrier of the Pt/BEFO film by the piezoelectric potential, and this enhanced (or reduced) the barrier does suppress (or enhanced) the photovoltaic effect of the heterostructure. Such a stable and distinct electric-field- and light-driven ferroelectric photovoltaic effect in the heterostructures may provide a pathway toward multistate memory and electro-optical devices.

To understand this behavior, it is noted that Fu *et al.* systematically investigated the relationship between the bandgap (*E*_g_) and the in-plane stress (*σ*_xx_) for the BFO film, and it was found that the *E*_g_ as a function of *σ*_xx_ can be described by the following relation:^33^

2 *E*_g_ = 2.71 + 0.67*σ*_xx_,

From eqn (2), the electric-field-induced shift in bandgap Δ*E*_g_ can be written as:

3 Δ*E*_g_(*E*) = 0.067[*σ*_xx_(*E*) − *σ*_xx_(0)],

where *σ*_xx_(0) and *σ*_xx_(*E*) are the in-plane stress of the BFO film under zero and nonzero electric field (*E*) applied to the PMN-PT substrate. For the BEFO film, the in-plane stress can be estimated using the biaxial strain model:^47^

4 *σ*_xx_(*E*) = *σ*_xx_(0) + *Yd*_eff_*E*,

where *Y* is the Young's modulus of the BEFO film, *d*_eff_ is the effective piezoelectric coefficient. We have:

5 *Y* = *c*_12_ − *c*_11_(*c*_11_ + *c*_12_)/2*c*_12_,

where *c*_*ij*_ denotes the elastic stiffness constants and one can choose *c*_11_ = 302 GPa and *c*_12_ = 162 GPa.^52^ Assuming a plane stress condition, one can have *d*_eff_ as6
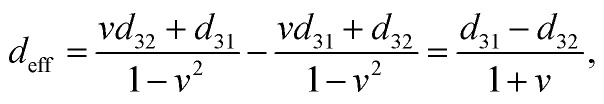
where *d*_31_ = −1750 pC N^−1^ along [100] direction and *d*_32_ = 900 pC N^−1^ along [01−1] direction for the (011) cut PMN-PT,^47^*v* is the Poisson ratio of the BEFO film. The piezoelectric strain-induced variation of bandgap *E*_g_, Δ*E*_g_, is estimated to be ∼0.036 eV. The decrease in the bandgap implies the higher possibility for electrons in the valence band to be activated onto the conduction band under the light illumination. It is believed to be one of the mechanisms responsible for the enhanced photovoltaic effect.

**Fig. 6:**
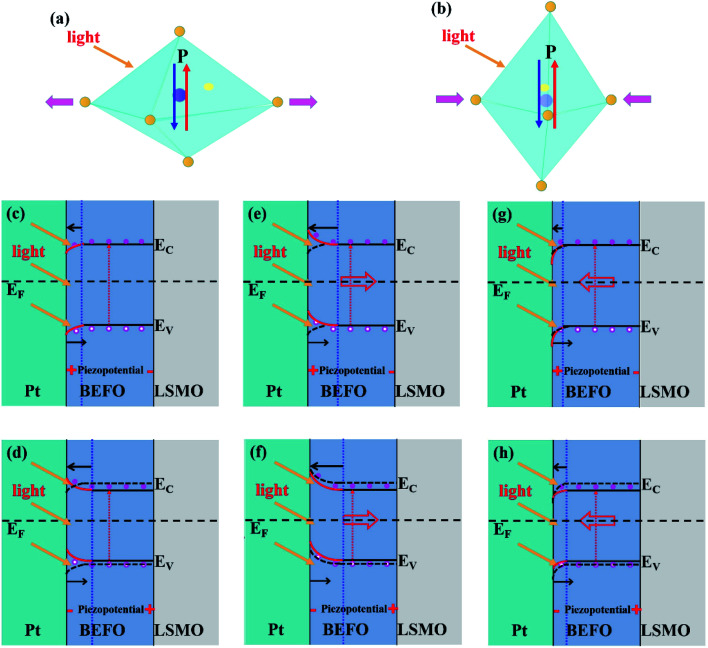
(a and b) show the schematic of the mutual interaction between the polarization switching, the light and the piezoelectric strain. Schematic of the energy band diagrams and principle of the photovoltaic properties for the Pt/BEFO/LSMO/PMN-PT heterostructure under in-plane compressed strain: (c) unpoled, (e) downward and (g) upward polarization states of BEFO; under in-plane tensile strain: (d) unpoled, (f) downward and (h) upward polarization states of BEFO, respectively. *E*_F_, *E*_V_, and *E*_C_ represent Femi level, valence band, and conduction band, respectively.

To further understand the electro- and mechano-driven photovoltaic effect, we sketch a schematic of the multi-field coupling and interfacial band diagrams in Fig. 6. In order to determine the energy band alignment of BEFO, we carried out ultraviolet photoelectron spectroscopy (UPS) measurement (the results are shown in Fig. S2†). The band-gap and electron affinity of BFO are 2.8 eV and 3.39 eV respectively.^53^ The work function of LSMO and Pt are 4.7 eV and 5.3 eV.^54^ Thus, a Schottky barrier can be developed at the Pt/BEFO interface, while the BEFO/LSMO interfacial band is nearly flat. On the other hand, due to lattice mismatch-induced residual stress, the fresh BEFO film is in the compressive strain state. This results in the positive and negative polarization charges created at the Pt/BEFO and BEFO/LSMO barriers. It is the reason why the self-polarization of the fresh BEFO film was oriented upwards.

The piezoelectric potential can be generated, which can modulate effectively the carrier transport and subsequently the energy band at the interface. The so-called piezo-phototronic effect, which causes the interfacial band bending *via* piezoelectric strain, can also modulate the interfacial carrier transport behavior. Under the light illumination, the photo-generated carriers are excited from the BEFO layer, and then separated by the net built-in electric field (*E*_bi_) which is the summation of the built-in field (*E*_in_) based on the Pt/BEFO Schottky barrier, the piezoelectric field *E*_piez_ induced by piezoelectric polarization charges, and the depolarization field *E*_dp_ caused by ferroelectric polarization.

It is well known that the ferroelectric polarization plays a dominant role in the transport of the metal/ferroelectrics/metal structure. For the BEFO with the downward polarization, the depletion region at the Pt/BEFO interface becomes wider and the band bending goes up. The direction of *E*_bi_ is upward aligned, generating a negative photocurrent and a positive photovoltage. On the other hand, for the BEFO film with the upward polarization, due to the positive polarization charges, the depletion region at the Pt/BEFO interface becomes narrower and the band bending goes down. The direction of *E*_bi_ is downward aligned, generating a positive photocurrent and negative photovoltage.

If the polarization is reversed, the direction of built-in field in the Pt/BEFO interface can be reversed. Therefore, the signs of *J*_sc_ and *V*_oc_ are opposite for the two different polarization states in the Pt/BEFO/LSMO/PMN-PT heterostructure (as shown in Fig. 3(b)). Moreover, the higher Schottky barrier at the Pt/BEFO interface provides a larger built-in field as a driving force to separate the photo-generated electron–hole pairs more efficiently. Therefore, the larger magnitudes of *V*_oc_ and *J*_sc_ in the Pt/BEFO/LSMO/PMN-PT heterostructure with the upward polarization were observed experimentally.

The modulation of the photocurrent by strain can be also discussed following this scheme. If a strain is applied, a certain amount of negative or positive piezo-charges will gather at the Pt/BEFO and BEFO/LSMO barriers, respectively. These piezo-charges can adjust the height and width of the Pt/BEFO barriers. When the direction of *E*_piez_ is parallel to *E*_dp_, the transport of photon-generated holes to the bottom of the BEFO layer will be enhanced. If the direction of *E*_piez_ is anti-parallel to *E*_dp_, the mobility of photon-generated holes would be suppressed.

Meanwhile, if the BEFO thin film is submitted to a compressive strain, the reduced *E*_g_ in BEFO will result in the release of more electrons from oxygen vacancies to the conduction band under the UV light illumination, leading to the enhanced carrier density. Conversely, if the BEFO film is in the tensile strain state, the enlarged *E*_g_ in BEFO reduces the carrier density. Given that tensile and compressive strains result in opposite electric field in a piezoelectric material, as negative and positive piezo-potential is applied to the BEFO film. Consequently, a permanent and controllable internal electric field can be introduced to reversibly modulate the height of potential barrier and width of depletion region at the Pt/BEFO interface by piezo-phototronic effect and ferroelectric polarization effect. Such an electric–optical–mechano driven photocurrent in a single device with diverse functionalities may provide a pathway toward multi-state memory and electro-optical devices.

## 4. Conclusions

In summary, we have successfully achieved an electro–opto–mechano-driven reversible multi-state memory based on photocurrent in the BEFO/LSMO/PMN-PT heterostructures. Here the key mechanism is that the competition between the built-in field (*E*_in_) based on the Pt/BEFO Schottky barrier, the piezoelectric field *E*_piez_ induced by piezoelectric polarization charges, and the depolarization field *E*_dp_ caused by ferroelectric polarization plays a key role in determining the modulation of the photovoltaic process. By electrically switching the polarization state between P_up_ and P_down_, the polarization direction can be detected non-destructively *via J*_sc_ at a zero-bias. Moreover, if a unit cell in P_up_ (or P_down_) state, its logic state will be alternately switched to On or OFF state when the incident light is on or off, and can be read out by detecting the corresponding *J*_sc_. Further, the piezoelectric potential can be generated when the BEFO in a compressive or tensile state, which can modulate effectively the carrier transport and subsequently the energy band at the interface. Based on the ferroelectric photovoltaic effect and piezo-phototronic effect, we achieved a five *J*_sc_ states in a unit cell, which is more beneficial for the multi-bit data storage and data readout. Meanwhile, it is interesting to note that the EOM_↑_ and EOM_↓_ can be as large as ∼1130% and 1200%, respectively, representing the largest values so far reported in literature. Our findings offer a new insight for integrated with much new functionality into one single device, which provide a pathway toward multi-state memory and electro-optical devices.

## Conflicts of interest

There are no conflicts to declare.

## Supplementary Material

RA-010-D0RA00725K-s001
