# Supplementary material for: Electro–opto–mechano driven reversible multi-state memory devices based on photocurrent in Bi0.9Eu0.1FeO3/La0.67Sr0.33MnO3/PMN-PT heterostructures
Source: RSC Adv. 2020 Apr 22;10(27):15784–93. doi: 10.1039/d0ra00725k (PMC9052503; doi:10.1039/d0ra00725k)
Supplement: RA-010-D0RA00725K-s001 [file RA-010-D0RA00725K-s001.pdf]

Electronic Supplementary Material (ESI) for RSC Advances.
This journal is © The Royal Society of Chemistry 2020

Supplementary Information

Electro-opto-mechano driven reversible multi-state memories based on photocurrent in $Bi_{0.9}Eu_{0.1}FeO_3$/$La_{0.67}Sr_{0.33}MnO_3$/PMN–PT heterostructures

Maocai Wei[a,b,*], Meifeng Liu[b], Lun Yang[b], Xiang Li[b], Yunlong Xie[b], Xiuzhang Wang[b*], Zijiong Li[a], Yuling Su[a], Zhongqiang Hu[c], and Jun-Ming Liu[b,d]

[a]*School of Physics and Electronic Engineering, Zhengzhou University of Light Industry, Zhengzhou 450002, China*

[b]*Institute for Advanced Materials, Hubei Normal University, Huangshi 435002, China*

[c]*Electronic Materials Research Laboratory, Key Laboratory of the Ministry of Education & International Center for Dielectric Research, Xian Jiaotong University, Xian 710049, China*

[d]*Laboratory of Solid State Microstructures, Nanjing University, Nanjing 210093, China*

* *Address correspondent to E-mail:* maocai0306@163.com (M. Wei)
* *Address correspondent to E-mail:* xzwang@hbnu.edu.cn (X. Wang)

Magnetic hysteresis (M-H) loops measurement

It is important to remember that $Bi_{0.9}Eu_{0.1}FeO_3$ (BEFO) is a multiferroic material, in which the ferroelectric, ferromagnetic and ferroelastic orderings interact with each other. The coupling among these ferroic orderings provides us with multiple degrees of freedom for controlling the photovoltaic effect, and this can be used to endow a memory unit with much new functionality. The magnetic properties are shown in Figure S1 with a typical magnetization curve at room temperature for the BEFO/LSMO/PMNPT heterostructures with the magnetic field applied along the [100] and [01-1] direction of the heterostructures after different poling states of PMN-PT, respectively. In the poled state, the M-H curve moves to left and the shape is slightly changed, with the coercive field ($H_{C)}$ increasing about 1 Oe for the [100] and decreasing 4 Oe for the [01-1] direction, respectively. However, there is a small exchange bias shift (about 1.8 Oe for the [100] and 2 Oe for the [01-1]) and no shift of the hysteresis loop (exchange bias) is also observed after poling due to the thick of LSMO layers [1]. The M-H loops at different poling states have shown that the magnetization of the heterostructure can be manipulated by the electric field induced piezo-strain, which could be used as evidence for magnetoelectric coupling effect at room temperature. However, we may need further research to understand this effect in depth.

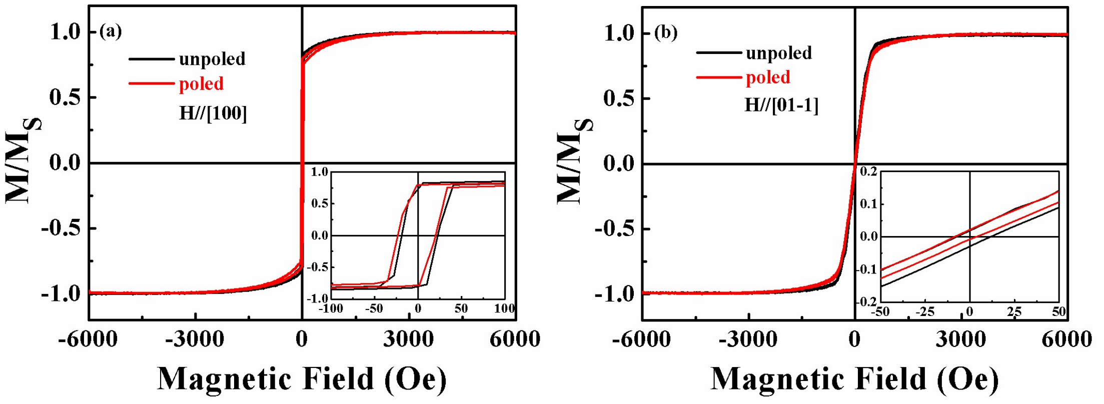

Figure S1 (a) and (b) show the M-H loops of the BEFO/LSMO/PMNPT heterostructures at different poling states with the magnetic fields along in-plane [100] and [100] directions, respectively, and the situation near zero field is amplified in the inset.

Ultraviolet photoelectron spectroscopy (UPS) measurement

Taking typical parameters W_{Pt} = 5.35 eV and W_{LSMO} = 4.96 eV. In order to determine the energy band alignment of BEFO, we carried out ultraviolet photoelectron spectroscopy (UPS) measurement (the results are shown in Figure S2). The Helium I radiation with a photon energy of 21.2 eV is used as the UV source. The work function (W_{BEFO}) calculated by subtracting the width of the spectrum from the photo energy is determined to be 4.59 eV. The energy level of the valance band, E_V, is 6.19 eV derived by extrapolating the linear portion of the low energy edge of the peak to energy axis (shown in the inset of Figure S2a). Considering that several works have reported that the direct band gap of the BFO films is below 2.8 eV [2-4], we take an integer of 2.8 eV for convenience. So the conduction band would be 3.39 eV. As shown in Figure S2b, the energy level diagram based on the UPS and analyzed results shows the valance, Femi and conduction energies band of the BEFO film.

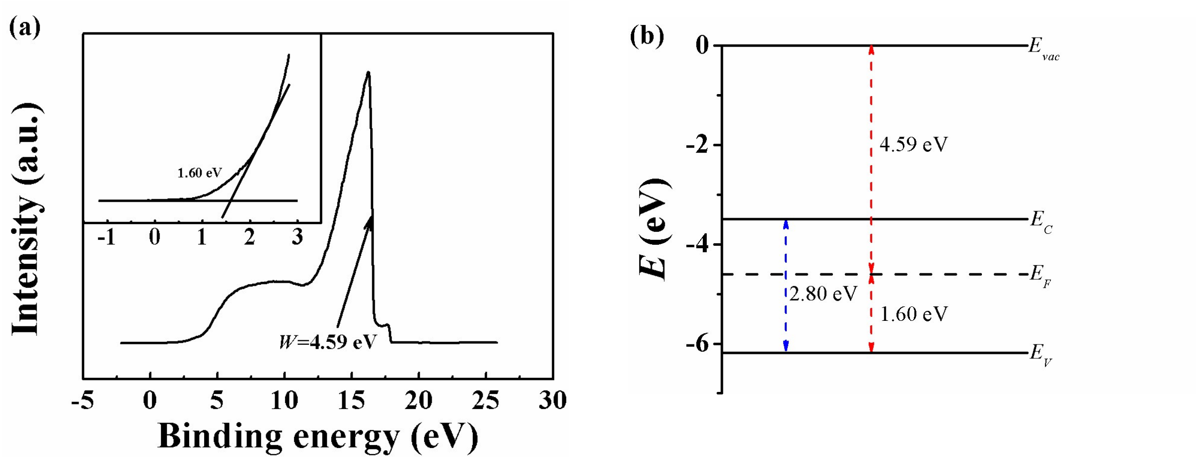

Figure S2 (a) UPS spectrum of the BEFO. Inset shows the enlarged spectra of low binding energy edge. (b) Energy level diagram showing the conduction, Femi and valence band energies of the BEFO.

References

1 M. Huijben, P. Yu, L. W. Martin, H. J. A. Molegraaf, Y. -H. Chu, M. B. Holcomb, N. Balke, G. Rijnders, and R. Ramesh, Adv. Mater. 2013, 25, 4739-4745.

2 W. Ji, K. Yao, Y. C. Liang, Adv. Mater. 2010, 22, 1763-1766.

3 D. Sando, C. Carrétéro, M. N. Grisolia, A. Barthélémy, V. Nagarajan, and M. Bibes, Adv. Optical Mater. 2018, 1700836.
4 D. Li, D. X. Zheng, C. Jin, W. C. Zheng, and H. L. Bai, ACS Appl. Mater. Interfaces 2018, 10, 19836-19843.
